# In Pursuit of Distinctiveness: Transmembrane Nucleoporins and Their Disease Associations

**DOI:** 10.3389/fonc.2021.784319

**Published:** 2021-12-14

**Authors:** Divya Bindra, Ram Kumar Mishra

**Affiliations:** Nups and SUMO Biology Group, Department of Biological Sciences, Indian Institute of Science Education and Research (IISER) Bhopal, Bhopal, India

**Keywords:** nucleoporins, POM121, NDC1, Nup210, cancer

## Abstract

The bi-directional nucleocytoplasmic shuttling of macromolecules like molecular signals, transcription factors, regulatory proteins, and RNAs occurs exclusively through Nuclear Pore Complex (NPC) residing in the nuclear membrane. This magnanimous complex is essentially a congregation of ~32 conserved proteins termed Nucleoporins (Nups) present in multiple copies and mostly arranged as subcomplexes to constitute a functional NPC. Nups participate in ancillary functions such as chromatin organization, transcription regulation, DNA damage repair, genome stabilization, and cell cycle control, apart from their central role as nucleocytoplasmic conduits. Thus, Nups exert a role in the maintenance of cellular homeostasis. In mammals, precisely three nucleoporins traverse the nuclear membrane, are called transmembrane Nups (TM-Nups), and are involved in multiple cellular functions. Owing to their vital roles in cellular processes and homeostasis, dysregulation of nucleoporin function is implicated in various diseases. The deregulated functioning of TM-Nups can thus act as an opportune window for the development of diseases. Indeed, mounting evidence exhibits a strong association of TM-Nups in cancer and numerous other physiological disorders. These findings have provided much-needed insights into the novel mechanisms of disease progression. While nucleoporin’s functions have often been summarized in the disease context, a focus on TM-Nups has always lacked. This review emphasizes the elucidation of distinct canonical and non-canonical functions of mammalian TM-Nups and the underlying mechanisms of their disease association.

## Introduction

The membrane encircling the nucleus is studded with Nuclear Pore Complexes (NPCs), facilitating the regulated mixing of nucleocytoplasmic contents. NPCs are large (~120MDa) assemblages of 30-32 pore-forming proteins called nucleoporins (Nups), exhibiting eightfold radial symmetry across the central core and a two-fold quasi-symmetry across the nuclear envelope (NE) (1, 2). While NPCs form a gateway, the nucleoporins act as the gatekeepers for molecular traffic and regulate NPC channels (3). The overall structural framework of NPC is fundamentally conserved across eukaryotic species; however, their size and Nup composition vary (4). While the nucleocytoplasmic transport of smaller molecules is passive, the directionally-regulated, selective, yet swift translocation of larger (>40kDa) cargos requires interaction with Nuclear Transport Receptors (NTRs). The bulk of nucleocytoplasmic transport (NCT) through the aqueous NPC pore utilizes karyopherin family NTRs (2, 5).

**Figure 1:**
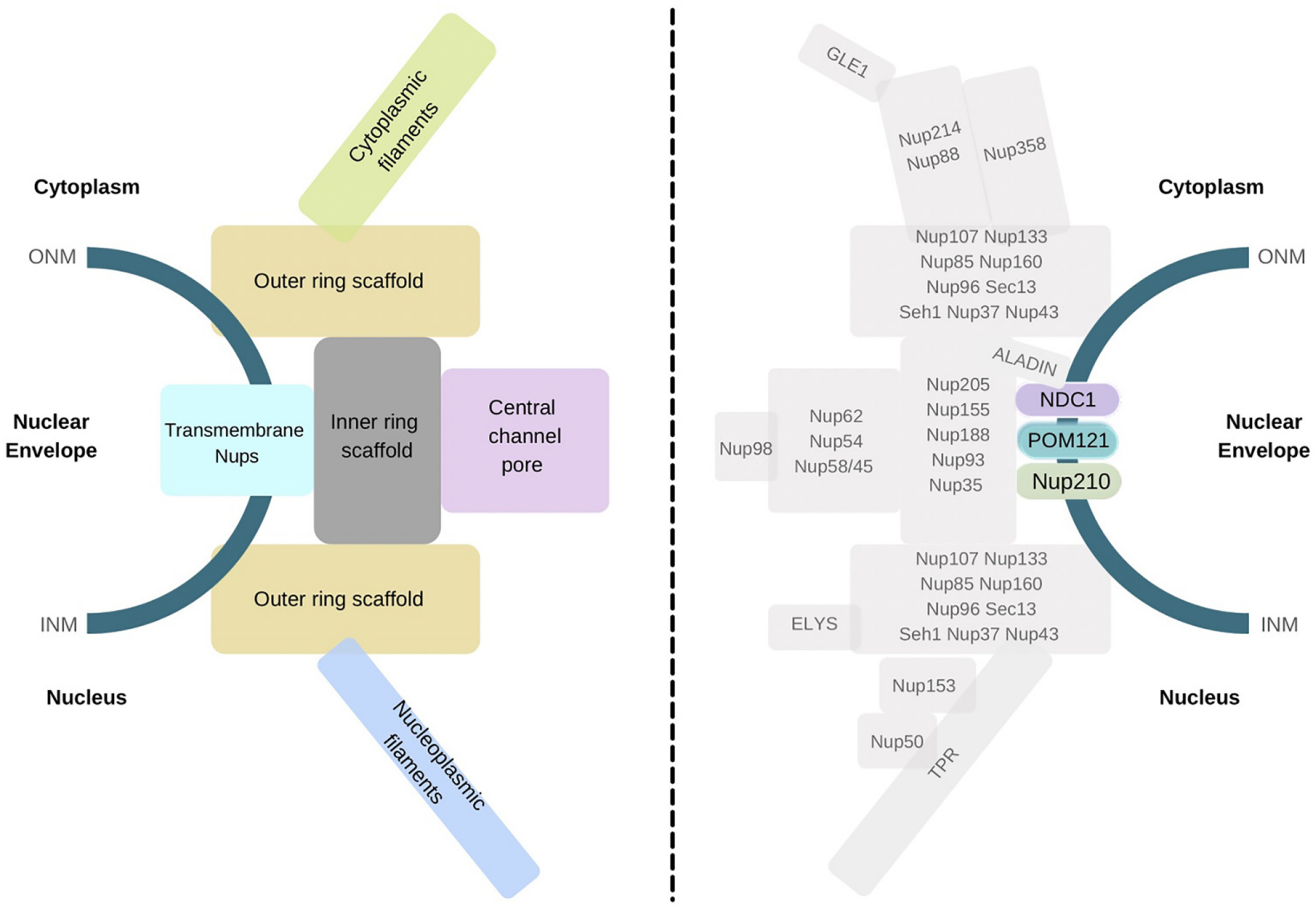
Schematic representation of NPC sub-complexes and constituent nucleoporins. A snapshot representation of nucleo-cytoplasmic face distribution of NPC sub-complexes across the nuclear membrane (left) and localization of individual Nups at the NPC (right). The distinct transmembrane Nups, POM121, NDC1, and Nup210 are highlighted and color-coded (right).

Nucleoporins are assembled and intricately arranged within the NPC as distinct and stable subcomplexes. All eukaryotic NPCs carry a central scaffold skirted by cytoplasmic and nucleoplasmic peripheral filaments. In mammals, the core scaffold coat is the unstructured FG repeat (-FxFG- and -GLFG-) domain-containing Nups, Nup62, Nup54, and Nup58/Nup45, which enclose the central channel and facilitate NTR mediated transport. The Nup93-Nup155 subcomplex forms the inner ring, and the symmetrically located core coat Nup107-160 subcomplex forms the outer rings. Nup88, Nup214, Nup358, Gle1, and ALADIN nucleoporins form the cytoplasmic filament subcomplex, while Nup50, Nup153, and TPR constitute the nuclear basket (6) (**Figure 1**). The magnanimous assembly of NPC requires tethering in the double-layered NE and is fastened there *via* attachment of membrane-spanning nucleoporins. Intriguingly in mammals, only three transmembrane nucleoporins (TM-Nups), POM121 (Pore membrane protein of 121kDa), NDC1 (Nuclear-Division-Cycle 1), and Nup210 (also called gp210), carry the onus of securing the large NPC at the NE. Of these, POM121 displays long residence time and low exchange rates at the NPC and is stable protein, whereas Nup210 displays shorter NPC residence time, indicating its non-structural functioning (7).

Post-mitosis, vesicles carrying these TM-Nups help initiate the nuclear membrane and nuclear pore reassembly (8). Canonically, these TM-Nups were considered as only structural elements of NPC; however, recent functional investigations on TM-Nups have uncovered their miscellaneous non-canonical functions like other Nups accomplished *via* dynamic properties exhibited by them. This review provides an account of hitherto under-appreciated functions and a perspective of evident disease association of TM-Nups.

## NPC Is a Conglomerate of Nucleoporins With Multifarious Activities

Nucleoporins, the structural and functional entities of NPCs, play critical roles in NCT (5, 9). For instance, the direct interaction of Nup62 with nuclear transport factor 2 (NTF2) regulates nuclear pore permeability and selective cargo shuttling across the NE, thus explicating a canonical function for the NPC (10, 11). However, nucleoporins are now well recognized for their additional non-canonical functions. Accordingly, nucleoporins participate in gene expression regulation (12, 13), DNA repair (14, 15), chromatin organization (13, 16), heterochromatin localization (17, 18), chromosome segregation (19), modulation of cellular signaling pathways (20, 21), cell differentiation and development (22–24). Apart from being static NPC components, Nups form dynamic entities as well. For example, Nup62 shuttles between the plasma membrane and the perinuclear compartment in HeLa cells, connecting Nup62 with cell migration (25). Similarly, the intranuclear functioning of Nup98 regulates gene expression (26, 27). Several nucleoporins localize outside the NPC and exhibit a plethora of transport-independent functions, also facilitating cell specificity. Moreover, the nucleoporin composition of the NPC itself displays tissue-type or cell differentiation stage-specific variations (22). The idea of NPC heterogeneity and specialization correlating with different transport-specific and tissue-specific functions has been previously proposed (28). Likewise, chromatin tethering of Nup93, Nup62, and Nup155 is ascribed in transcriptional regulation to direct the genome expression profile of the cell, thereby enabling cell specificity (29). Nup155 exerts epigenetic control over cardiac cell growth through interaction with histone deacetylase 4 (HDAC4) to modulate specific gene expression (30). Interestingly, differential expression of Nup62 in the prefrontal cortex (31) and CA3 hippocampal region (32) of the brain correlates with chronic stress and depression. Such observations have highlighted an extensive range of functions adopted by the nucleoporins critical for proper cellular functioning.

Given the diverse and tissue-specific properties of nucleoporins, it is no surprise that a perturbation in their structure-function and protein levels induces a myriad of physiological disorders. Contextually, identifying aberrations in Nup coding genes underpins numerous diseases like autoimmune defects, neurological complications, and cancers. Primarily, chromosomal translocation events have led to the formation of chimeric fusion proteins. Accordingly, Nup214/CAN and tyrosine kinase ABL1 chimera formation induced gene expression dysregulations causing elevated expression of oncogenesis promoting genes (33). Similarly, mutations and altered expression of Nups frequently cause Nup-mediated disease pathophysiology and are extensively observed in several carcinomas (34–36). Nuclear pore components such as Nup62 and Nup153 also affect TGF-β and Wnt/β-Catenin cellular signaling pathways that are now being comprehended (37, 38). Interestingly, the TM-Nups have also been recognized to encompass diverse roles, including transcription regulation. A detailed mechanistic understanding of the individual Nup or NPC functioning can aid in the development of mitigation strategies for Nup-associated diseases (or nucleoporopathies).

## Dynamic Cellular Functions of the Transmembrane Nucleoporins

The TM-Nups constitute only those Nups that tether the central framework of the pore complex to the NE. Attempts to assess the role of TM-Nups in NPC assembly and anchoring identified only two Nups, POM121, and Nup210 initially. Both were suggested to be crucial for nuclear membrane fusion and assembly of the pore complexes. POM121 plays a role in NPC congregation and docks the NPC to NE at the double membrane fusion pore. While NPC assemblage at the interphase requires POM121, it is dispensable for the inner (INM) and outer nuclear membranes (ONM) fusion during post-mitotic events (39–41). The distinct NE-binding region, determined using live imaging, allows POM121 to localize to the INM components like lamin B receptor (LBR) during the initial seeding steps of NPC assembly (39). Interactions of the nuclear localization signals (NLSs) of POM121 with importin-α and importin-β aid in its active nuclear targeting or import during interphase (39). A report simultaneously provided evidence for transient association of POM121 with another INM protein, Sun1 (cytoskeleton interacting protein), which localizes to forming NPCs at interphase (42).

Furthermore, during NPC assembly, the N-terminal region of POM121 associates with two scaffold subcomplexes by directly interacting with Nup155 and Nup160, members of the Nup93 and the Nup107-160 subcomplex, respectively (43). These observations provided insights on how POM121 anchors NPCs to NE and the integration of the Nup107-160 complex as one of the rapid early events of interphase NPC assembly. Although classified as immobile and fixed TM-Nup, the remarkable discovery of a soluble form of POM121 or sPOM121 reflected on its intranuclear regulatory role. sPOM121 isoform, encoded from an alternate transcription start site, lacks the N-terminal transmembrane domain and NPC localization. Thus, the intranuclear off-pore functions of sPOM121 are exerted by co-binding with Nup98, aiding the retention of Nup107-160 complex components in the nucleoplasm and altogether regulating transcription at the gene promoters (44).

Structurally, Nup210 is required during NPC disassembly and NE breakdown (45). Nevertheless, the proposed role for Nup210 in the process of NPC assembly was challenged when Nup210 exhibited cell-type-specific expression during mouse organogenesis. Nup210 gene and transcript expression was revealed to be very low or absent in many developing mouse tissues and dividing cell lines (46). While Nup210 is absent in undifferentiated cells, it is expressed during myogenesis or differentiation of embryonic stem cells and functions independent of its NPC association (47). Additionally, analysis of Nup210 deficient cells showed that POM121 and Nup107 could remain steadily associated with the NPC in its absence (48). Nonetheless, myogenic differentiation is dependent on Nup210 mediated recruitment of the Mef2C transcriptional complex at the nuclear periphery, which results in the induction of differentiation-specific gene profiles (49). These observations indicated the dispensable nature of Nup210 in NPC assembly and tethering along with its tissue-specific and dynamic functioning beyond NPC structural recruitment and maintenance. However, Nup210 has been ascertained to regulate gene expression and induce genes needed for cell differentiation (21). For mouse fibroblast differentiation into neural stem cells (NSCs), Nup210 functions to activate the SoxB1 family of transcription factors (50). Nup210 has also been found essential for T-cell homeostasis and modulates TCR signaling (51, 52).

The reasonable extent of NPCs assembled in human cells lacking membrane-spanning Nups POM121 and Nup210 (53) prompted the identification of additional factors required for NPC assembly. Already recognized as a cell cycle modulator in budding yeast (54), NDC1 was later characterized as a part of the NPC constituting one of the TM-Nups in the vertebrates (55). NDC1 interacts with Nups like Nup53 and plays critical roles in NPC assembly and anchoring into the NE (56, 57). NDC1 also anchors another nuclear pore protein ALADIN onto the NE (58), and this interaction is linked with disease pathophysiology as discussed henceforward.

## Molecular Mechanisms Underlying Transmembrane Nup-Mediated Diseases

Growing evidence has underscored the association of mammalian TM-Nups in several physiological diseases, including cancer. However, the molecular mechanisms associated with transmembrane Nup-mediated disease development are yet to be uncovered conclusively. Nevertheless, various studies have elucidated the genetic and molecular roles of individual TM-Nups that lead to numerous disease pathologies summarized in **Table 1**. Here, we discuss each one of them in the context of physiological anomalies that their loss induces.

**Table 1:** List of transmembrane nucleoporins-associated diseases and suggested molecular mechanisms.

Transmembrane Nucleoporin	Disease	Underlying Molecular Mechanism/Defect(s)
**POM121**	Prostate cancer	POM121 upregulation, Nuclear import of transcription factors MYC, E2F1, AR, GATA2 (59)
	Acute Lymphoblastic Leukemia	Gene translocation and fusion with PAX5 (60, 61)
	Amyotrophic Lateral Sclerosis/Frontotemporal Dementia	Deregulation of other Nups, NCT defects (62)
	Non-Small-Cell Lung Cancer	Modulation of TGF-β/SMAD and PI3K/AKT pathways (63)
	Oral Squamous Cell Carcinoma, Laryngeal Cancer, Colorectal Cancer	POM121 upregulation (64–66)
	Inflammation	P65 transport inhibition, NFκB pathway repression (67)
**NDC1**	Ischemic Cardiomyopathy, Dilated Cardiomyopathy, Non-Small-Cell Lung Cancer	Upregulation of NDC1 (68, 69)
	Esophageal Squamous Cell Carcinoma	mRNA transport anomaly (70)
	Non-Small-Cell Lung Cancer	Apoptotic pathway modulation (69, 71)
	Cervical Cancer	Wnt/β-catenin pathway modulation (72)
	Triple-A syndrome	Interaction with and recruitment of ALADIN (58)
	Infertility	NDC1 mutation, Interaction with regulatory molecule Septin12 (73, 74)
**Nup210/gp210**	Primary Biliary Cholangitis	Nup210 upregulation, autoantibody-mediated heightened immunoreactivity (75, 76)
	Prostate Cancer	Nup210 upregulation, Androgen receptor (AR) splice variant-7 (AR-V7) mediated activation (77)
	Liver Cancer	Nup210 upregulation, scaffold for SMARCB1 chromatin remodeler binding (78)
	Lung Cancer	H3K27ac and H3K4me3 histone modifications (79)
	Cervical Cancer	Nup210 upregulation, miR-22-NUP210-Fas axis modulation (80, 81)
	Amyotrophic Lateral Sclerosis	Age-dependent mislocalization and precipitation with Nup205 at NE (82)
	Focal cerebral ischemia	Mislocalization of Nup210 with Nup205 (83)
	Endometriosis	Rs354476 polymorphism within *NUP210* gene affecting miRNA hsa-miR-125b-5p binding site (84)

### POM121

POM121 is encoded by two different gene loci (designated locus A and C) on chromosome 7q11.23 to form POM121A and POM121C in humans (85). POM121C has been linked to body mass index (BMI), particularly in monogenic obesity syndromes (86). Abdominal adipose tissue biopsies highlighted a role for POM121C in type-2 diabetes-related insulin resistance (IR) by stimulating adipogenesis and increasing adipocyte sensitivity to insulin (87). However, further comprehensive scrutiny is necessary to establish its role in controlling systemic sensitivity to insulin and other metabolic traits. Population-based fluorescence *in situ* hybridization (FISH) analysis demonstrated chromosomal translocation events involving the *POM121* gene forming gene fusions leading to cancer. POM121 fusion with PAX5 (a transcription factor critical in B-cell development) generates aberrant chimeric proteins in childhood acute lymphoblastic leukemia (ALL) (60). Detailed analysis of PAX5 and POM121 gene translocation revealed that genetic rearrangements between chromosomes 7, 9, and 12 produce the in-frame chimeric protein (N-terminal PAX5 DNA binding paired domain merges with POM121) with higher probable occurrence in pediatric ALL than adult ALL (61). Additionally, the PAX5-POM121 fusion protein localizes to the nucleus, where it may also stimulate PAX5 target genes such as CD79A, likely indicating transcription activation (61). This observation highlighted an alternate role of the structural nucleoporin POM121 in transcription regulation. Apart from acting as a gene fusion product, POM121 also contributes to cancer progression by mediating the transport of oncogenic molecules into the nucleus. While POM121 is highly upregulated in advanced lethal prostate cancer (PC), it is found to promote PC aggressiveness by augmenting the selective importin-dependent nuclear shuttling of oncogenic MYC, E2F1, Androgen receptor (AR), and GATA2 (PC-specific) transcription factors (TFs) (59). In agreement, inhibition of the POM121-importin-β axis is shown to result in reduced tumorigenicity and proliferation in pre-clinical models, representing this as a potential pharmacological target in lethal PC (59). POM121 has also been linked as a potential prognostic biomarker in oral squamous cell carcinoma (OSCC) (64), colorectal cancer (CRC) (65), laryngeal cancer (LRC) (66), and non-small-cell lung cancer (NSCLC) (63), where elevated levels of POM121 are linked to advanced tumor-node-metastasis (TNM) stages. A recent study on POM121 in NSCLC has provided insights into the involvement of TGF-β/SMAD and PI3K/AKT signaling pathways in cancer cell proliferation and metastasis (63). However, this is just the beginning of our understanding of cellular pathways modulated by TM-Nups.

Interestingly, nuclear transportation is utilized by POM121 to exert an immune-modulatory effect on macrophage inflammation by repressing the NFκB pathway and inhibiting phosphorylated p65 protein nuclear translocation (67). The mechanistic underpinnings of C9orf72 in cellular toxicity and ultimately neuronal degeneration dependent Amyotrophic Lateral Sclerosis and Frontotemporal Dementia (ALS/FTD) emphasized the pathologic effects of POM121 depletion downstream of G4C2 repeat RNA expression. Super-resolution microscopy of spinal neurons revealed that the deregulation of POM121 resulted in the consequent decline of other Nups in the nucleus, thereby causing NCT defects and cellular toxicity in ALS/FTD (62). The molecular basis in C9orf72-mediated ALS may also potentially reflect similar pathology of NCT disruption and abnormal build-up of NPC-associated proteins as detected in multiple neurodegenerative diseases.

### NDC1

NDC1 (also known as Transmembrane Protein 48 or TMEM48) anchors another nuclear pore protein ALADIN onto the NE, while a mutation in the latter disrupts this interaction (58). ALADIN mutations are also shown to result in triple-A syndrome, thus providing indirect evidence that NDC1 may be linked to the development of this disorder. The study of mutations in NDC1 in mice has been shown to cause defective gametogenesis, infertility, and skeletal deformities. NDC1 forms complex with Septin12 (*SEPT12* gene product is necessary for imparting sperm morphological characteristics) and affects its localization during murine spermiogenesis, highlighting the involvement of NDC1 in sperm head and tail development (73, 74). NDC1 is upregulated and mislocalized into the nucleus in ventricular cardiac tissues of ischemic cardiomyopathy and dilated cardiomyopathy (ICM and DCM, respectively) (68). Such alterations may reflect defective nucleocytoplasmic trafficking and nuclear organization in ventricular cells of the heart, but the exact molecular mechanism for progression towards heart failure (HF) is yet to be defined.

Altered expression of NDC1 is also seen in the development of numerous malignancies. Accordingly, it was found overexpressed in NSCLC cell lines, H1299, and A549 (69). Downregulation of NDC1 inhibits DNA replication and cell cycle-associated genes like *PCNA* and *CYCLINB1* and reduces cell proliferation and migration. Moreover, studies in nude mice revealed that NDC1 inhibition decreases cell migration and tumorigenicity and induces apoptosis (69). Thus, suppression of NDC1 might render a potential therapeutic effect against NSCLCs. Indeed, another study evaluating the effect of miRNA-induced silencing of NDC1 in lung cancer demonstrated similar outcomes. Significant suppression of NDC1 in A549 cells by miR-421 leads to the augmented expression of tumor suppressors and pro-apoptotic molecules like PTEN, Caspase3, and TP53, thereby inducing apoptosis (71). These results indicate that NDC1 modulates the apoptotic pathway, albeit the precise apoptotic regulation needs further speculation. In a gene enrichment and meta-analytic study, *NDC1* was reported as one of the critical genes in addition to *NUP107* and *NUP155* genes regulating esophageal squamous cell carcinoma (ESCC) (70). These nucleoporin genes were attributed to the RNA transport pathway; nonetheless, their mechanistic role in ESCC progression requires substantiation. A study examining the signal transduction pathway influenced by NDC1 in cervical cancer (CC) reported that NDC1-mediated cell proliferation and metastasis is modulated partly by activating the Wnt/β-catenin pathway (72). The downregulation of NDC1 in CC cell lines (HeLa and SiHa) curtailed the levels of β-catenin (effector of Wnt pathway) and its targets T cell factor 1 (TCF1) and axis formation inhibitor 2 (AXIN2) and reduced cell growth in grafts (*in vivo*). In contrast, activator-mediated induction of the Wnt pathway could significantly reverse the effect of NDC1 knockdown on cell proliferation and migration in CC. This observation emphasizes the downstream contribution of the Wnt pathway in cancer progression as a result of NDC1 alterations.

### Nup210

Gene-specific expression studies identified Nup210 or gp210 as one of the prime targets for autoantibodies in primary biliary cholangitis (PBC), an autoimmune disease of the liver (75). Consequently, it was also overexpressed in the biliary epithelial cells of the liver small bile duct in PBC patients, wherein it depicts enhanced immunoreactivity (76). Analysis of Nup210 in tumorigenesis shows its upregulation in several carcinomas as the basis of cell proliferation, including cervical cancer (CC) (80, 81), lung carcinoma (LC) (79), and prostate cancer (PC) (77). Nup210 has also been determined as an epigenetic biomarker in lung adenocarcinoma as the gene promoter region undergoes H3K27ac and H3K4me3 histone modifications (79). Studies inspecting the nature of Nup210 function in cancer progression have described diverse molecular mechanisms moderated by this transmembrane protein. Upregulated Nup210 in HeLa cells is attributed to the downregulation of miR-22, as miR-22 normally binds with Nup210 transcript and inhibits its expression.

Moreover, miR-22-Nup210 deregulation results in apoptotic inhibition *via* regulation of Fas expression, thereby affecting Fas signaling. Thus, the miR-22-Nup210-Fas axis is suggested in CC progression (81). Nup210 has also been shown to interact with and act as a scaffold for chromatin remodeler SMARCB1 (fundamental subunit of the SWI/SNF chromatin remodeling complex) in liver cancer (78). Nup210-SMARCB1 interaction mediates the oncogenic effects demonstrated by the upregulation of this remodeling protein. In PC cells, the *NUP210* gene is specifically targeted downstream of androgen receptor (AR) splice variant-7 (AR-V7) activity which drives the progression of primary PC to castration-resistant prostate cancer (CRPC) (77). Mislocalization of Nup210 has also been commonly observed in cerebral ischemic tissues and neurodegenerative Amyotrophic lateral sclerosis (ALS), wherein Nup210 co-localizes and forms precipitate with Nup205 (82, 83). The role of Nup210 has also been examined in endometriosis. Here, a genetic modification in the 3’-UTR of *NUP210* gene, i.e., Rs354476 polymorphism (miRSNP or SNP at microRNA binding site), affects its binding with hsa-miR-125b-5p, a microRNA critical in the development of endometriosis (87).

**Figure 2:**
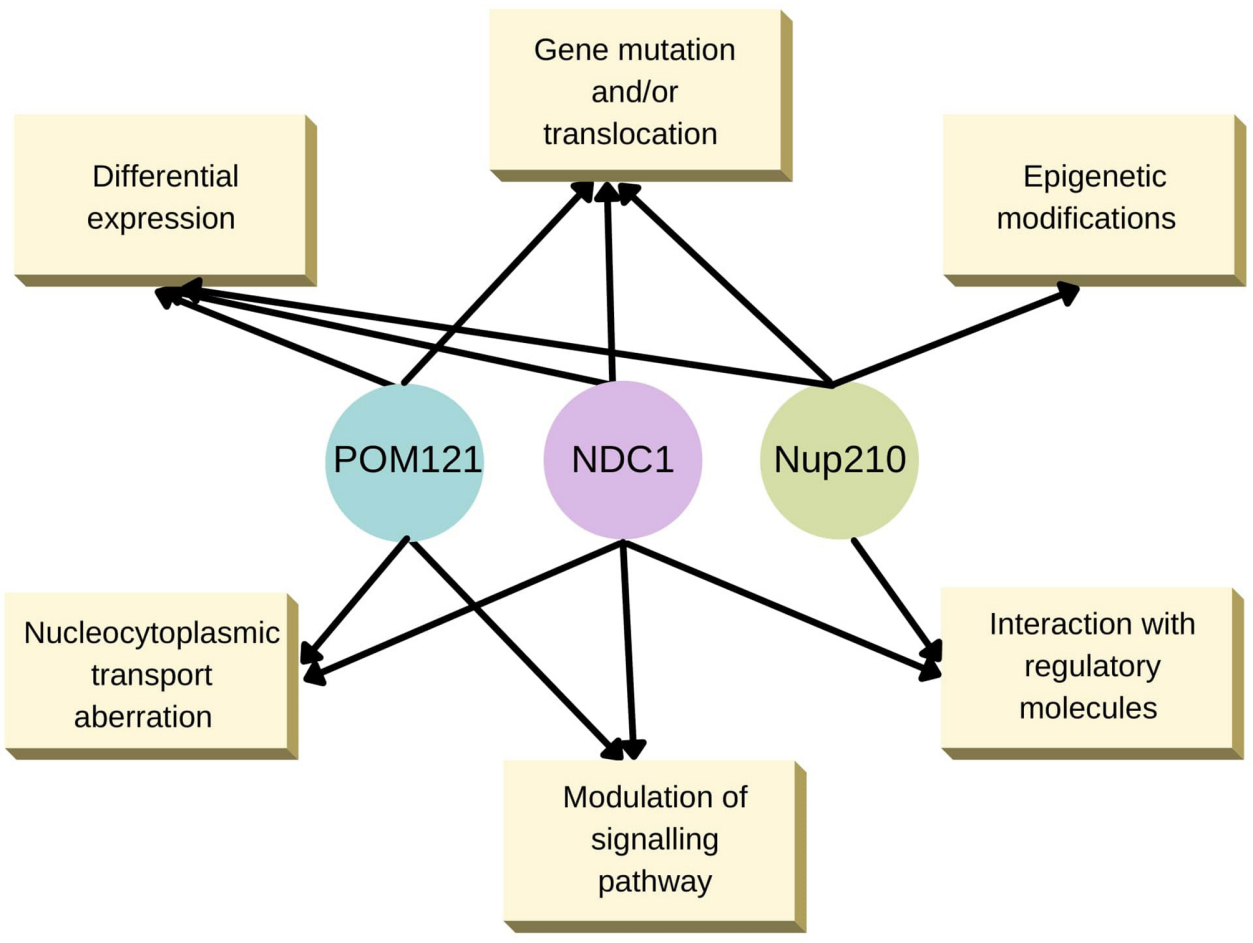
Known transmembrane Nup alterations underlying human diseases. An interaction web of the three TM-Nups, POM121, NDC1 and Nup210 that regulate various cellular processes.

The intermolecular interactions mediated by various TM-Nups affecting several cellular processes are summarised in **Figure 2**. It is easily conceivable from the summary that TM-Nups play a critical role in cellular homeostasis but are undermined by the overwhelming presence of their non-TM cousin Nups. Given the value these TM-Nups hold by sheer location in the cells, they can be a prominent player in cellular events directly affected by perturbations in strict nucleocytoplasmic partitioning. The interplay of TM and non-TM Nups becomes very important in this context and needs urgent attention in physiologically relevant health conditions.

## Conclusion

Thus far, studies evaluating the three known mammalian TM-Nups, POM121, NDC1, and Nup210, have illuminated their functional plasticity and distinctiveness by regulating functions like nuclear envelope assembly, nuclear pore insertion in NE, nucleocytoplasmic transport, and gene expressions. Nevertheless, specific molecular interactions and mechanisms in such diseases and identification of other diseases associated with TM-Nups are worth exploring. Although an association is made between TM-Nups and disease, the molecular details await identification and recognition. The lack of structural information also impedes the desired progress. Therefore, further structural, biochemical, and functional analyses are necessary to realize the functional significance and diverse roles of TM-Nups. Various cellular signaling pathways where TM-Nups participate are beginning to emerge, and their incipient mechanistic examinations in disease conditions continue to build a platform for in-depth analysis. Intriguingly, the tissue-specific expression of such TM-Nups also invokes undertaking an engaging analysis of their tissue-specific variations and any association with disease outcomes. We envisage that research groups will undertake a more focussed approach to uncover the relevance of TM-Nups in a myriad of cellular processes, tissue specificity, and disease association, including but not limited to organ-specific disease, and cancer.

## Author Contributions

DB and RKM developed the idea, collected information, and wrote and edited the manuscript. RKM supervised manuscript development and arranged the funding. All authors contributed to the article and approved the submitted version.

## Funding

RKM was supported by the SERB (CRG/2020/000496) grant, intramural funding, and infrastructural support from IISER Bhopal.

## Conflict of Interest

The authors declare that the research was conducted in the absence of any commercial or financial relationships that could be construed as a potential conflict of interest.

## Publisher’s Note

All claims expressed in this article are solely those of the authors and do not necessarily represent those of their affiliated organizations, or those of the publisher, the editors and the reviewers. Any product that may be evaluated in this article, or claim that may be made by its manufacturer, is not guaranteed or endorsed by the publisher.
